# The Role of Non-Mycorrhizal Fungi in Germination of the Mycoheterotrophic Orchid *Pogoniopsis schenckii* Cogn.

**DOI:** 10.3389/fpls.2019.01589

**Published:** 2019-11-29

**Authors:** Laís Soêmis Sisti, Denisele Neuza Aline Flores-Borges, Sara Adrián López de Andrade, Samantha Koehler, Maria Letícia Bonatelli, Juliana Lischka Sampaio Mayer

**Affiliations:** ^1^Laboratory of Plant Anatomy, Department of Plant Biology, Institute of Biology, State University of Campinas, Campinas, Brazil; ^2^Laboratory of Plant Molecular Physiology, Department of Plant Biology, Institute of Biology, State University of Campinas, Campinas, Brazil; ^3^Laboratory of Plant Taxonomy, Department of Plant Biology, Institute of Biology, State University of Campinas, Campinas, Brazil; ^4^Laboratory of Genetics of Microorganisms, Department of Genetics, College of Agriculture “Luiz de Queiroz,” University of São Paulo, Piracicaba, Brazil

**Keywords:** endophytic fungi, symbiotic germination, plant anatomy, aclorophyllated plant, ultrastructure, protocorm, Orchidaceae, Tulasnellaceae

## Abstract

Endophytic fungi are those that inhabit within organs and tissues without causing damage, while mycorrhizal fungi develop hyphal complexes called pelotons within cortical cells of orchid roots. Although abundant and frequent in all plant organs, the role of endophytic fungi has been neglected in relation to orchid’s early development. *Pogoniopsis schenckii* Cogn. is an aclorophyllated and mycoheterotrophic (MH) orchid. This study aimed at i) investigating the endophytic fungal community in organs of *P. schenckii* and its mycorrhizal fungi associated; ii) evaluating the ability of isolated fungus in the *in vitro* germination of the seeds of the species, and iii) describing the development of *P. schenckii* protocorm, analyzing the ultrastructure of the infected cells. Six genera of fungi were isolated and identified through the partial sequencing of the internal transcribed spacer region, all belonging to the phylum Ascomycota. Also, Tulasnellaceae was identified through uncultured technique as potentially mycorrhizal in this MH orchid. Some isolates of the genera *Trichoderma*, *Fusarium*, and especially *Clonostachys* presented germinative potential on *P. schenckii* seeds, causing rupture of the external tegument. The protocorms showed complete absence of peloton formation, but fungal hyphae were clearly observed within living cells. This is the first report of germination of a MH and aclorophyllated orchid species stimulated by the presence of non-mycorrhizal endophytic fungi isolated from fruits and roots of the same species.

## Introduction

Mycoheterotrophic (MH) plants evolved independently in several locations and represent one of the most extreme forms of mycorrhizal dependence (Leake, 1994). These plants remain aclorophyllated throughout their life cycle and are totally dependent on their mycorrhizal partners for their survival (Peterson et al., 2004). There are approximately 235 MH species within the Orchidaceae family (Merckx, 2013). One of the main characteristics of mycorrhizal orchids is the formation of a hyphal complex, also called peloton, which develops within the parenchyma cells of the roots and rhizomes of such plants (Peterson et al., 2004; Rasmussen and Rasmussen, 2009). During the germination in *Orchidaceae*, the embryo swells and promotes the seed coat rupture, thus forming a cone-shaped structure (Arditti, 1967), also known as protocorm (Bernard, 1909, qt. in Leroux et al., 1997). This structure is considered an intermediate phase between the embryo and the seedling (Leroux et al., 1997). After seed coating rupture, absorbent trichomes appear, the protocorm increases in size, the apical meristem is installed, and the first leaves are formed, all that followed by the development of adventitious root (Arditti, 1967; Harrison, 1977).

Under natural conditions, even photosynthetic orchids undergo a phase of mycorrhizal fungi dependence at their early stages of growth and development, not being able to germinate in their absence (McKendrick et al., 2000; Leake et al., 2004). Some species of green orchids can be germinated *in vitro*, in an asymbiotic way, if soluble carbohydrates and other organic compounds are supplied (Rasmussen and Rasmussen, 2009). Otherwise, it is necessary to establish a symbiotic association with an appropriate fungal partner and a complex carbohydrate source (Zettler, 1997; Peterson et al., 2004). During the symbiotic germination, anatomical analyzes allows the observation of pelotons in protocorm cells of MH species, and transmission electron microscopy is important to investigate and confirm that plant cells containing hyphae remain alive and with intact organelles, ensuring that the association with the fungus is not harmful to the plant. In addition, the latter allows verifying if the hyphae have clamp connections, dolipores, and parenthesomes, typical characteristics of Basidiomycota. As described by Roy et al. (2009), in MH orchid *Epipogium aphyllum*, the authors observed, in electron micrographs, the presence of clamp connections, dolipores, and parenthesomes, and confirmed the identification of *Inocybe*, a basidiomycetous symbiont, by means of molecular techniques.

Orchids can relate to a great diversity of fungal taxa and combine it with different nutritional strategies (Rasmussen, 2002; Peterson et al., 2004). Photosynthetic orchid species are generally associated with rhizoctonia-like fungi group belonging to the families Ceratobasidiaceae, Tulasnellaceae, and Sebacinaceae (Otero et al., 2002; Rasmussen, 2002). However, the heterotrophic species of temperate regions often associate with ectomycorrhizal basidiomycetes, as those of the families Thelephoraceae and Russulaceae (Julou et al., 2005; Roy et al., 2009). In addition, MH species from tropical regions appear to exhibit greater diversity in their symbiotic associations and less degree of specificity when compared with species from temperate regions, associating with predominantly saprophytic fungi (Martos et al., 2009; Selosse et al., 2010). It has been shown in a study by Yagame et al. (2007), which revealed the association of the Asiatic orchid *Epipogium roseum* with saprophytic fungi belonging to *Psathyrella* or *Coprinus* in Coprinaceae, isolated from root and rhizome. Even though most MH species belong to tropical regions, most of the studies include species belonging to temperate regions as models (Selosse et al., 2010).

Although mycorrhizal associations with orchids are predominantly related to the fungi of the phylum Basidiomycota, groups of Ascomycota have already been described forming such association. In a previous work by Selosse et al. (2004), it was possible to verify the formation of pelotons by fungi belonging to the order Pezizales, previously identified as ascomycetes, present in root cells of the green *Epipactis microphylla*, analyzed by light and transmission electron microscopy. According to the authors, an endophyte was found forming this association with the orchid, and it belongs to a fungal group known as ectomycorrhizal in tree roots. Endophytic microorganisms, unlike pathogens, do not cause damage to the host and are known for establishing beneficial or neutral associations with plants (Petrini, 1991). They inhabit the internal part of organs and tissues of plants, and can confer protection against pathogens, or cause the production of plant growth factors (Azevedo, 1998; Ma et al., 2015). Although their role in orchids is rarely addressed, non-mycorrhizal endophytic fungi can be found in all organs of the plant, encompassing more than 110 genera, predominantly belonging to the phylum *Ascomycota* (Ma et al., 2015).

The genus *Pogoniopsis* belongs to the subfamily Vanilloideae, Pogoniae tribe (Cameron, 2009). It is composed only of two species, *Pogoniopsis schenckii* Cogn. and *Pogoniopsis nidusavis* Rchb.f. & Warm., and both are MH. Both develop under organic matter in dense tropical and subtropical forests, are aclorophyllated, and have a pale-yellow coloration. The plants of the genus have short and fasciculate roots, with bracts that cover the floral stem. *P. schenckii* is a poorly-known species, with collections described in several states of Brazil (Bittencourt and de Gasper, 2016). Despite being widely distributed, it is considered a rare species, and it is included in the Red List of the Threatened Flora of Paraná State (1995), and classified as a vulnerable species by the Red List of Threatened Flora of São Paulo State, both in Brazil (2004) (CNCFlora, 2012). In addition, in a recent study, projections on future climate changes indicated that with global warming, the species *P. schenckii* could have its ideal niche reduced by up to 30% of its current extent (Kolanowska et al., 2017), severely compromising its survival. Therefore, studies to understand its germination and biology, as well as strategies to enable its establishment *in vitro*, can be of great value for the conservation of populations of this species.

In a previous analysis, it was possible to observe the presence of fungal hyphae inside the mature fruits of *P. schenckii* (data not shown), and this finding promoted the hypothesis that these fungi could be related to the germination of the species, in addition to those present in the roots. Moreover, there is lack of studies on the endophytic fungi present in other organs of MH orchids and not only in the roots, especially when dealing with their function for germination of these plants (Ma et al., 2015). Therefore, other organs of the species (floral stem and fruits) were also included in the studies of isolation and identification of fungi in order to test such hypothesis. This work was generally aimed at i) investigating the endophytic fungal community of *P. schenckii* and the non-cultivable mycorrhizal fungi present in their roots; ii) evaluating the role of these fungi in germination of the seeds of *P. schenckii*, and iii) monitoring the development of the protocorm after germination.

## Material and Methods

### Vegetal Sampling Collection

**Figure 1:**
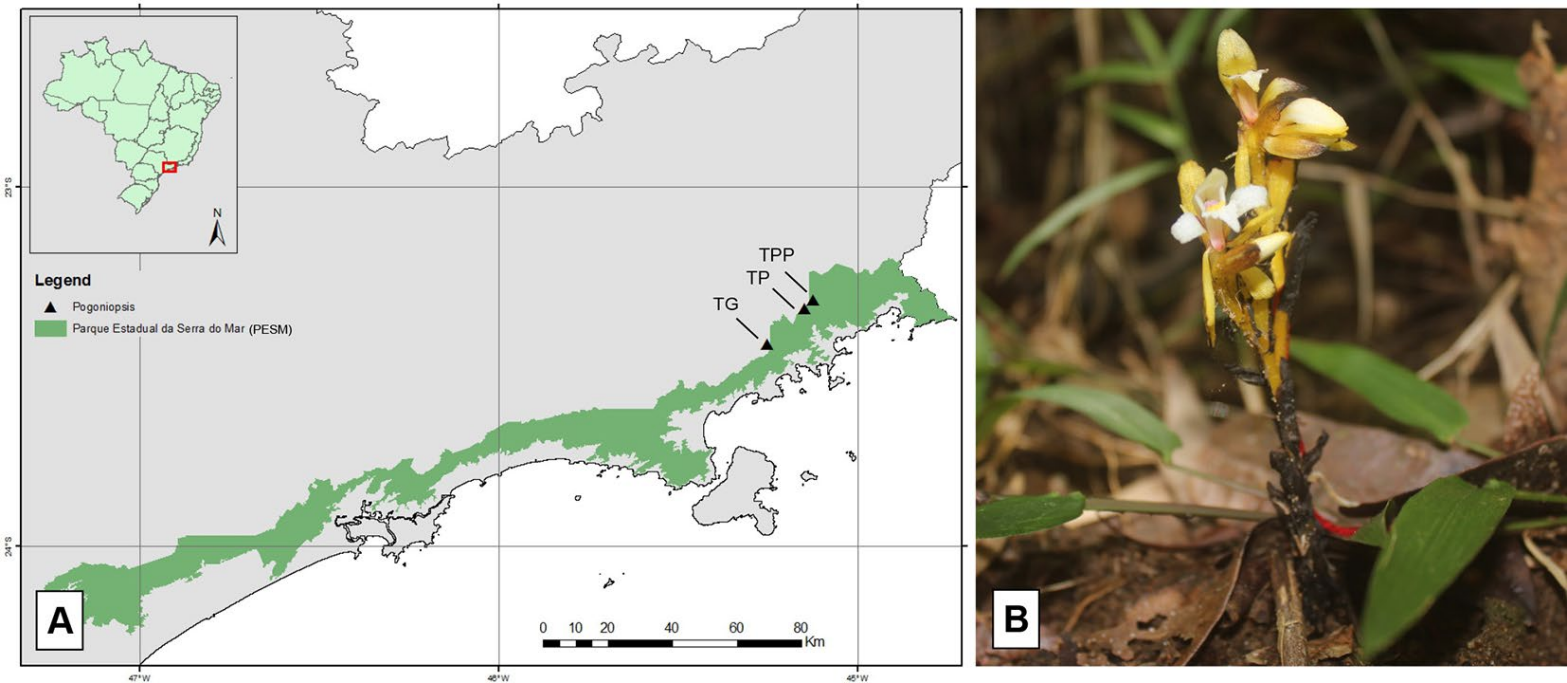
Representation of the Serra do Mar State Park **(A)** (in green) and location of the trials (triangles) where *Pogoniopsis schenckii* **(B)** individuals were collected.

Roots, fruits, and floral stems samples of *P. schenckii* were collected from three populations located at three trails: *Trilha do Poço do Pito* (TPP), *Trilha do Pirapitinga* (TP), and *Trilha do Garcez* (TG) (**Figure 1**). All trails belong to the Santa Virgínia Nucleus (SVN), located in the Serra do Mar State Park, in the outskirts of the municipalities of São Luiz do Paraitinga and Natividade da Serra, São Paulo State, Brazil. Access to the study area and collection permission was authorized by COTEC through permission (291/2018 D83/2018 PM) and São Paulo State Environment Secretariat (SMA) permission 260108 – 005.510/2014.

### Isolation of Fungi and Analysis of Frequency of Isolation

In order to investigate the community of endophytic and mycorrhizal fungi of *P. schenckii*, roots, floral stems, and fruit portions of the three populations were isolated according to the methodology described by Araújo et al. (2001). Healthy plant samples were washed in running water, and then washed for 1 min with 70% ethanol solution, followed by a 3 or 5 min commercial sodium hypochlorite (2% active chlorine) washing, and a 30 s 70% alcohol washing. Finally, two washes were performed in autoclaved distilled water, and an aliquot of the water from the last wash was plated in commercial PDA (potato dextrose agar) medium to verify the efficacy of surface disinfection. After, fragments of about 5 mm were sectioned and inoculated in PDA with addition of ampicillin (75 µg/ml) and left to grow at room temperature in the dark for 5 days. After the incubation period, the grown hyphae were striated in WA (water agar: 7 g/L agar) and kept at room temperature and in the dark. After 3 days, fragments of the WA containing the grown fungal mycelium were inoculated at the center of PDA plates and incubated for 7 days under the same conditions previously described. The isolated fungi were placed in mineral oil and stored.

In order to calculate the frequency of isolation (FI) of fungi, the calculation described in Araújo et al. (2014) was performed, which consists in evaluating the number of plant fragments that presented fungal growth in relation to the total number of fragments sampled.

### Total Deoxyribonucleic Acid Extraction and Identification of Isolated Fungi

For identification of the isolated fungi, the methodology of total fungal DNA extraction described in Raeder and Broda (1985), with modifications by Araújo et al. (2014), was used. The fungi were inoculated in PDA and grown for approximately 10 days at room temperature. The grown fungal mycelium was scraped from the surface of the culture medium, crushed in liquid nitrogen, and approximately 200 mg of the crushed mycelium was transferred to a 2 ml tube. Then, 1 ml of extraction buffer (1% SDS, 25 mM EDTA, 250 mM NaCl, and 200 mM Tris-HCl [pH 8.0]) was added to the tube, vortexed, and incubated at 65°C for 20 min. The solution was centrifuged at 10,000 xg for 10 min at 4°C and the supernatant was transferred to a new tube containing 800 µl of phenol. The suspension was homogenized by inversion and centrifuged at 10,000 x*g* for 10 min at 4°C. The supernatant was transferred to a new tube and 400 µl phenol and 400 µl chloroform (1:1) were added. The solution was homogenized by inversion and centrifuged at 10,000 x*g* for 10 min at 4°C. The upper phase was transferred to a new tube containing 800 µl of chloroform, homogenized by inversion, and centrifuged at 10,000 xg for 10 min at 4°C. The supernatant was transferred to a new tube and 450 µl of isopropanol was added. The solution was homogenized by inversion, incubated for 5 min at room temperature and centrifuged at 10,000 x*g* for 5 min at 4°C. The supernatant was discarded and the precipitate (DNA) was washed with 500 µl of 80% ethanol and centrifuged at 10,000 xg for 5 min at 4° C. Then, the ethanol was discarded, the DNA was dried at 37°C for 30 min and finally resuspended in 40 µl of deionized distilled autoclaved water and kept in a refrigerator overnight. The DNA suspension was quantified in a spectrophotometer (NanoDrop 2000c, Thermo Scientific) and stored in a freezer at −20°C.

For amplification of the fragment of interest, amplification of the ITS1-5,8S-ITS2 region was performed using the primers ITS1 (5’-TCCGTACCTCAACCTGCGG-3’) and ITS4 (5’-TCCTCCGCTTATTGATATGC-3’) (White et al., 1990). Polymerase chain reaction (PCR) was performed with 3.7 mM MgCl_2_, 1 mM of each deoxynucleoside triphosphate, 0.4 µM of each primer, 2.5 U of Taq DNA polymerase, 1 X buffer, 5 ng DNA in a final volume of 50 µl. The ITS1-5.8S-ITS2 region of recombinant DNA (rDNA) was amplified by 24 cycles of PCR reaction with initial denaturation of 4 min at 94°C, followed by 24 cycles of 30 s at 94°C, 1 min at 55°C; 30 s at 72°C, and a final extension of 7 min at 72°C in thermal cycler (Peltier Thermal Cycler 200, MJ Research). The reaction product was analyzed by using agarose gel (1% w/v) along with the 1 Kb/100 pb DNA molecular weight marker (Invitrogen). Amplified (400 pb) rDNA ITS1-5, 8S-ITS2 region fragments were sequenced at the company Macrogen (Seoul, South Korea) and the sequences obtained were used for phylogenetic identification on the NCBI (National Center for Biotechnology Information— www.ncbi.nlm.nih.gov) database through Blastn.

**Figure 2:**
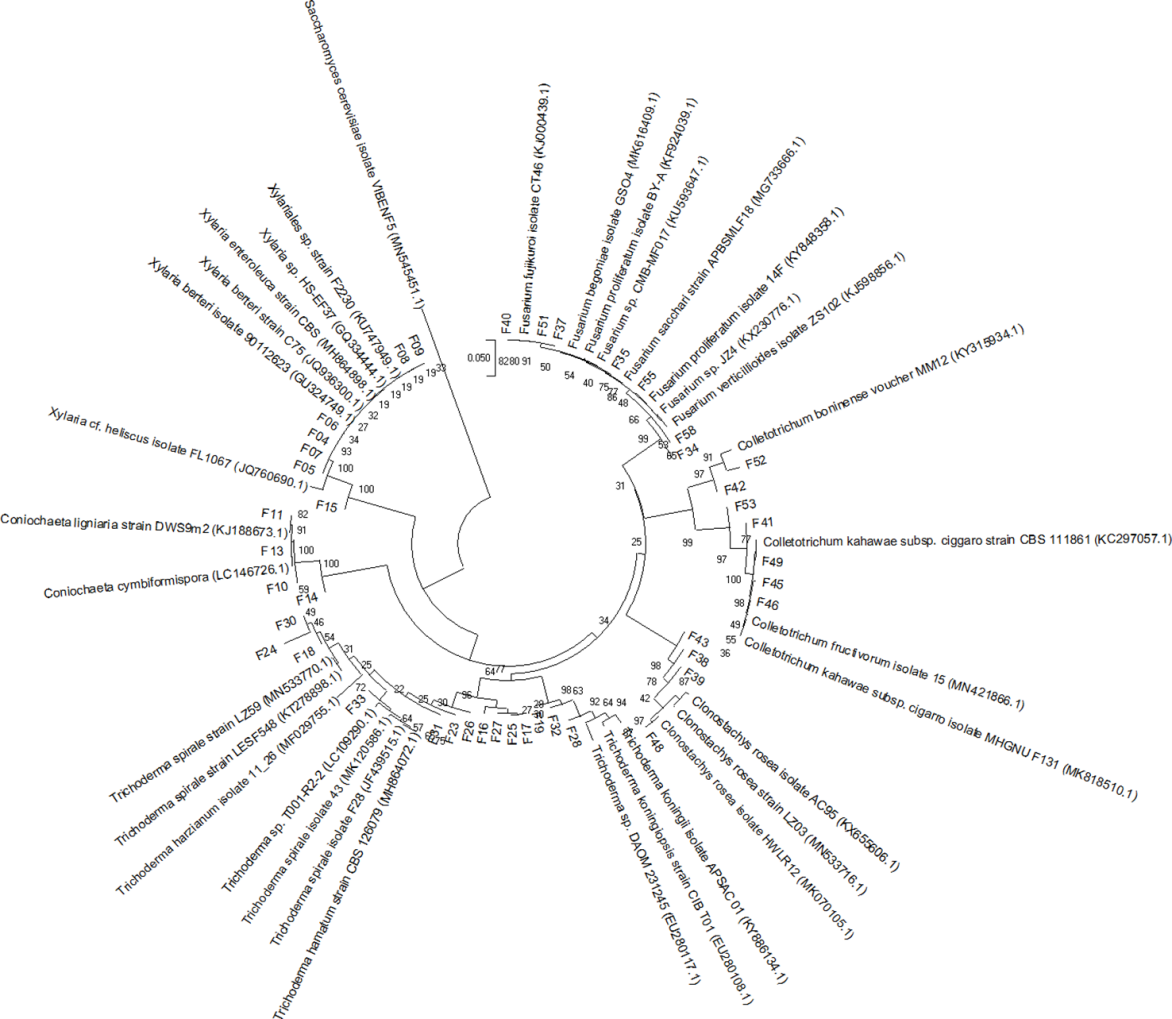
Neighbor-joining tree, based on Jukes and Cantor’s model, obtained from the analysis of recombinant DNA internal transcribed spacer sequences of 42 fungi isolated from *Pogoniopsis schenckii*. Reference sequences from GenBank were used to compare the relationships among the isolates. The Basidiomycota *Saccharomyces cerevisiae* (MN545451.5) was used as the out-group. Bootstrap values (n = 1,000 replicates) are shown at the intersections.

A phylogenetic tree was built using the MEGA 10.0 program (Kumar et al., 2018). The alignment was made with MUSCLE default settings. The evolutionary history was inferred according to the neighbor-joining method (Saitou and Nei, 1987), considering bootstrap test (1,000 replicates) (Felsenstein, 1985), and the evolutionary distances were computed using the Jukes-Cantor method (Jukes and Cantor, 1969) (**Figure 2**). Sequences were deposited in GenBank under the accession numbers MN611256 to MN611298.

### Total Root Deoxyribonucleic Acid Extraction and Identification of Basidiomycetes

Roots of *P. schenckii.* were used order to investigate the presence of Basidiomycetes that could not be cultivated. For this purpose, DNA extractions were made from root samples of TPP and TP populations, which were collected and promptly stored at −80°C. The methodology used was described in (Tel-Zur et al., 1999), with modifications. In this procedure, the samples were macerated and placed in a microtube, and then 1.5 ml of sorbitol (0,35 M sorbitol, 100 mM Tris-HCl, 5 mM EDTA) was added. The samples were vortexed, incubated in the refrigerator for 20 min, and centrifuged for 10 min at 10,000 x*g* at 4°C. The supernatant is discarded and 800 µl of cetyl trimethylammonium bromide and 30 µl of sakosyl 30% were added. The samples were then vortexed and incubated at 65°C for 1 h. Then 600 µl of chloroform-isoamyl alcohol was added and the samples were vortexed again and centrifuged for 10 min at 10,000 x*g*. The supernatant was placed in a new microtube with 30 µl of ammonia acetate and 500 µl of isopropanol, and the samples were incubated at −20°C overnight. In the next day, samples were centrifuged at 15,000 x*g* for 30 min at 4°C. The supernatant was discarded, 500 µl of 70% ethanol were added and centrifuged for 10 min at 15,000 x*g* at room temperature. The supernatant was discarded again, the samples were incubated at 65°C for 30 min, 50 µl of deionized distilled water is added, and the samples were incubated at 37°C for 2 h. The extraction products were analyzed with 1% agarose gel with the 1 kb molecular weight marker.

The amplification of the region of interest was performed through the primers ITS1-OF and ITS4-OF (Taylor and McCormick, 2008) and following the methodology indicated by the authors. An aliquot of the product of this first reaction was used in a second reaction for the primers ITS4-tul and ITS1, according to their recommendation. The amplified samples were sequenced at the company Macrogen (South Korea) and the sequences obtained were used for phylogenetic identification on the NCBI (National Center for Biotechnology Information— www.ncbi.nlm.nih.gov) database through Blastn.

### Symbiotic, Asymbiotic, and *In Situ* Germination

For symbiotic germination trials, fruits from two isolated populations of *P. schenckii* were superficially disinfected according to the methodology described in Pereira et al. (1993) and Araújo et al. (2001). After disinfection, the fruits were sectioned and the seeds removed. The seeds were disinfested by immersion in sodium hypochlorite (1% active chlorine) for 7 min and washed three times with autoclaved distilled water. The seeds were inoculated on filter paper fragments arranged on plates containing OMA-oatmeal agar (2 or 4 g/L oat flour and 7 g/L agar [pH 6.0]), as suggested by Zettler (1997). Each plate was inoculated with the mycelium of fungal representatives isolated from root or fruit of *P. schenckii*; the plates were sealed and incubated in the dark at room temperature. For control purposes, plaques with seeds were maintained in the same culture medium without inoculation of fungi. The seeds were transferred monthly to new plates with oat meal agar and analyzed periodically, and the development of the protocorm was classified in phases, according to their size.

For the asymbiotic germination, the same methodology of disinfestation of the seeds described for the symbiotic germination trial was used. The culture medium used was the MS, proposed by Murashige and Skoog (1962), with modifications. The concentration of micro and macro-nutrients was reduced by half and the medium supplemented with 30 g/L sucrose and 0.5 mg/L nicotinic acid, 0.5 mg/L pyridoxine HCl, 0.1 mg/L thiamine HCl, 2.0 mg/L glycine, and 100 mg/L myo-inositol (pH 6.0). The plates were sealed, incubated in the dark in a growth chamber at 27°C, and analyzed periodically.

For the *in situ* germination trial, a methodology proposed by Rasmussen and Whigham (1993) was used. Several mature fruits of a population of *P. schenckii* were placed in small packages made of nylon, whose pores allow the passage of water and nutrients and soil bacteria and hyphae of filamentous fungi. After being properly sealed, the packages were tied to a stake fixed to the ground and buried superficially under litter in the TPP site, near the same population of plants of their origin. The fruits buried were monitored and recovered periodically for seed germination analysis.

### Anatomical, Ultrastructure, and Surface Analysis

Samples of protocorms at different stages, as well as samples of buried field structures, were fixed with 2.5% glutaraldehyde in 3% sodium cacodylate buffer (pH 7.25) for 24 h at 4°C. Post-fixation was performed with 1% aqueous osmium tetroxide (OsO_4_) overnight. The samples were then washed three times in distilled water and dehydrated in a rising ethyl series. After dehydration, the samples were soaked in hydrophilic acrylic resin LR White^®^ Hard Grade (EMS) and polymerized in gelatin capsules in an oven at 60°C for 12 h. The ultrafine sections were contrasted with uranyl acetate (Watson, 1958) and lead citrate (Reynolds, 1963) and examined under a Philips EM 100 transmission electron microscope operated at 60 Kv in the Laboratory of Electron Microscopy of the Institute of Biology of UNICAMP.

For analyzes of the anatomical structures of the protocorms, as well as in the transmission electron microscopy analysis, samples of different sizes were processed, ultrafine sections were stained with 0.05% toluidine blue (Sakai, 1973) in phosphate and citrate buffer pH 4.5, and analyzed under a light microscope. To record the results, the images were captured using an Olympus DP71 video camera coupled to an Olympus BX 51 microscope.

To investigate the surface of the samples, protocorms in different phases and fixed in solution of Karnovsky (Karnovsky, 1965) were used. After fixation, the samples were dehydrated in ethyl series and dried by the CO_2_ critical point method in a Balzers CPD 030 Critical Point Dryer. The material was then mounted on metal supports and covered with colloidal gold for 220 s with a Bal-Tec SCD 050 Sample Sputter Coater. The analysis and electromicrographic recording were performed using a LEO VP 435 scanning electron microscope at 20 kV, at the Institute of Biology of UNICAMP.

## Results

### Isolation and Identification of Fungal Community From *Pogoniopsis schenckii* Organs

**Figure 3:**
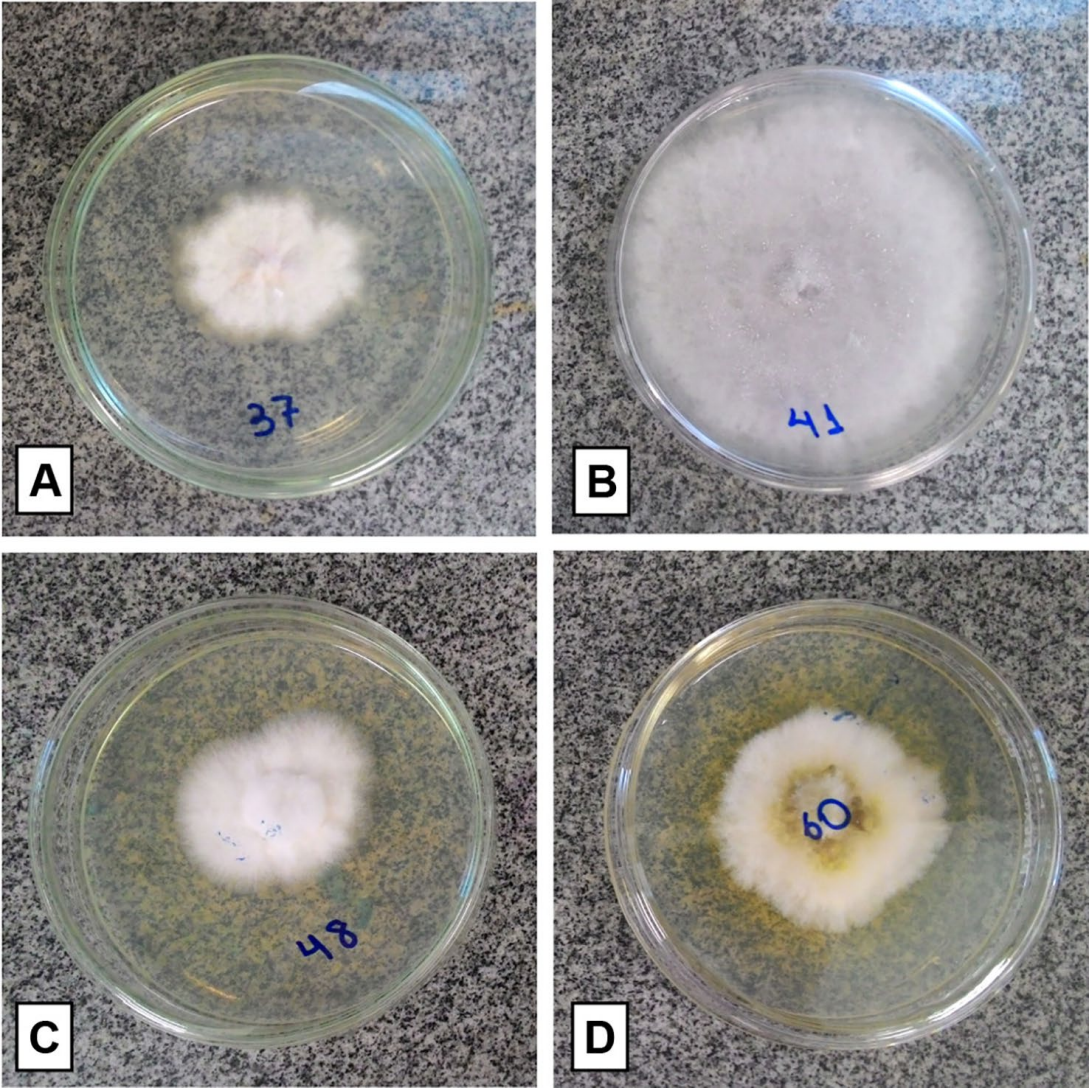
Isolated fungal representatives of different organs of *Pogoniopsis schenckii*. *Fusarium* sp. and *Colletotrichum* sp., respectively, isolated from fruit **(A**, **B)**. *Clonostachys* sp., both isolated from floral stem **(C**, **D)**.

Thirty-three fungi were isolated from the roots, 19 from the fruits, and 16 from the floral stems of *P. schenckii*. The fungal isolates were purified and stored, totalizing 68 fungi from all populations and different plant organs (**Figure 3**). None of the surface disinfestation methodologies generated fungal contamination in the water of the last wash. Forty-two isolates were identified, all belonging to the phylum Ascomycota. Among the fungi isolated from the roots, the genera *Xylaria* and *Coniochaeta* were found as endophytic in *P. schenckii* individuals from TG population, and only the genus *Trichoderma* were isolated from individuals of TP and TPP populations. As for floral stem and fruit portions of TP and TPP individuals, the genera *Fusarium*, *Clonostachys*, and *Colletotrichum* were reported in both organs. Further details on isolation and identification of the fungal community can be found in **Supplementary Table 1**.

A total of three isolation procedures were carried out at different stages of plant development. Initially, the plant material used for the isolation were root portions of two populations of *P. schenckii*, TG, and TP, through the modified surface disinfestation methodology of Pereira et al. (1993) and Araújo et al. (2001), aiming at increasing the efficiency of disinfestation (5 min in hypochlorite). However, this first trial resulted in the isolation of 15 fungi only from TG population, with isolation frequency (IF) of 12.5%. Therefore, in a second isolation, the authors’ methodology was used without modification (3 min in hypochlorite) for isolating fungi from the roots of the plant. In this experiment, FI of 38.8 and 41.6% were obtained for TPP population and TP population, respectively, both located in the SVN, and a total of 18 fungi were isolated from the *P. schenckii* populations. Due to the higher IF obtained in the second experiment, the unmodified methodology was adopted for a third isolation round. In this isolation, fruit and floral stem portions of NSV individuals were used without discriminating the populations, obtaining 100% IF for both organs.

### Identification of Non-Cultured Root Basidiomycetes

Identification of the sequences obtained from the roots of *P. schenckii* with the specific primers ITS4-tul and ITS1 revealed the presence of the fungus from genus *Tulasnella*, belonging to family Tulasnellaceae and Basidiomycota phylum.

### Germination Trials

In a first trial, beginning in April 2016, a total of 18 fungal representatives isolated from the roots and 13 fungi from the fruits of *P. schenckii* were selected according to their morphology to be inoculated in seeds of TP and TPP populations. After about 20 days of plate incubation, it was possible to observe the external seed coat rupture of both populations in the presence of two fungal isolates from the roots of five from the fruits. At 40 days of experiment, two other fungi, one of root and another of fruit, were able to cause the external seed coat rupture of both populations. However, among the total of nine fungi, three isolates of root, and six of fruit, only the isolates F38 and F39 caused visible changes in the development of the protocorms after the external tegument rupture, both fungi of fruits of *P. schenckii*. The experiment exceeded 15 months, presenting minimal changes only in the size of the protocorms, under magnifying glass. Among the fungi that showed to be effective in germination, they comprise the genera *Trichoderma* sp. (F16, F25, and F32), *Fusarium* sp. (F34, F37, and F40) and *Clonostachys* sp. (F38, F39, and F43).

**Figure 4:**
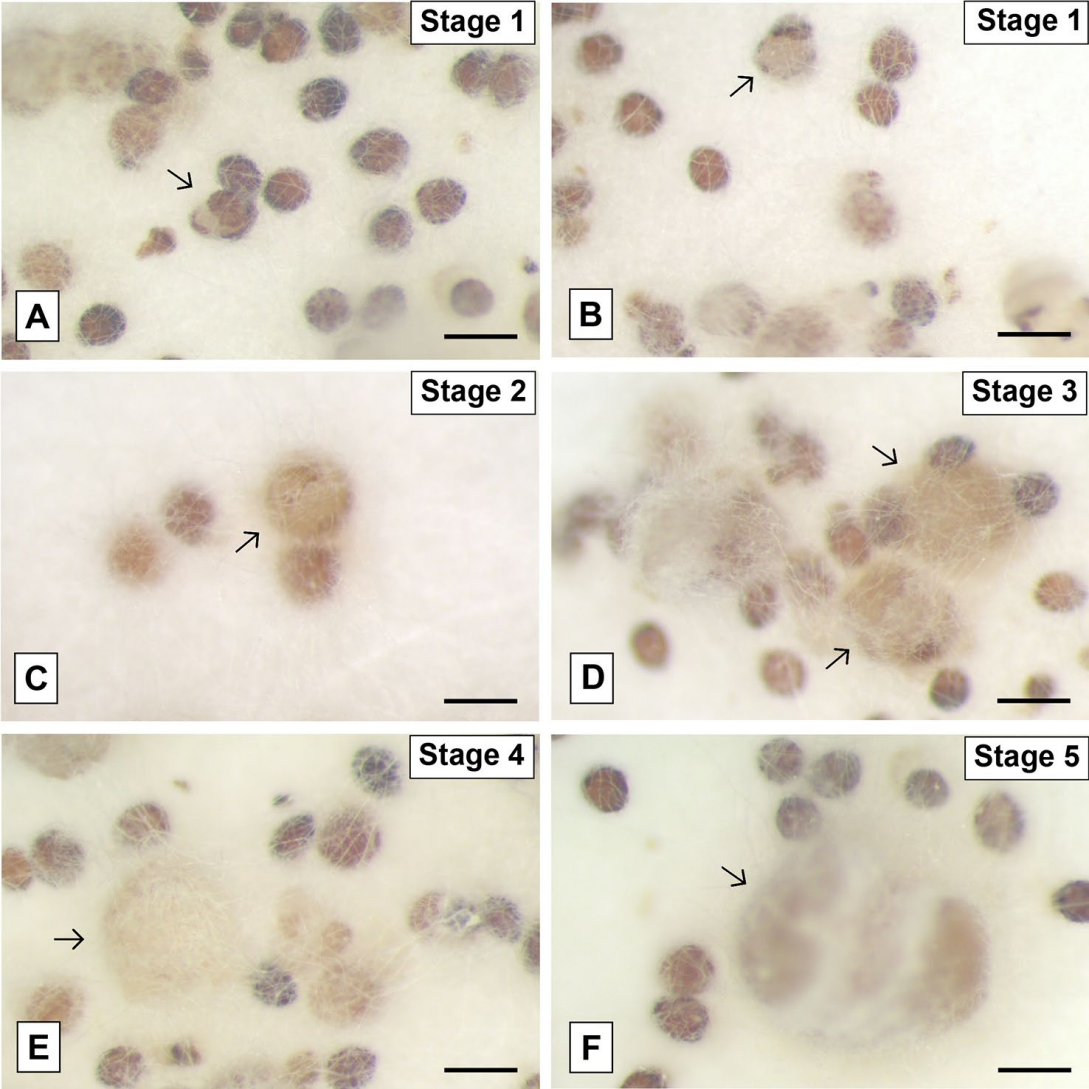
*Pogoniopsis schenckii* seeds inoculated with the fungal isolate of the genus *Clonostachys* (F38) isolated from *P. schenckii* at different stages of the germination. Seeds showing swelling and rupture of the external tegument **(A**, **B)** and subsequent growth and development of the protocorms (arrows) after testa rupture **(C**–**F)**. Scale bar—200 μm.

The mature seeds of *P. schenckii* had a rounded shape and they were slightly tapered at one end. Its external tegument is extremely rigid and dark brown in color (**Figure 4A**). At the initial stage of germination, the seed underwent a slight swelling accompanied by the whitening of the external tegument, almost imperceptible changes. After this stage, the testa rupture occurred (**Figures 4A, B**), and the protocorm began to increase in size (**Figures 4C–E**), at a very slow growth rate, assuming a slightly elongated shape (**Figure 4F**).

No changes were observed in the seeds during the whole period of incubation of the asymbiotic germination plates in MS medium, which took about 15 months, suggesting the inefficiency of germination in the absence of fungi and under such cultivation conditions. The same occurred with the sowing and seed recovery trials *in situ*, which did not demonstrate efficacy in the germination of seeds of *P. schenckii* either.

### Anatomical, Ultrastructure, and Surface Analysis

**Figure 5:**
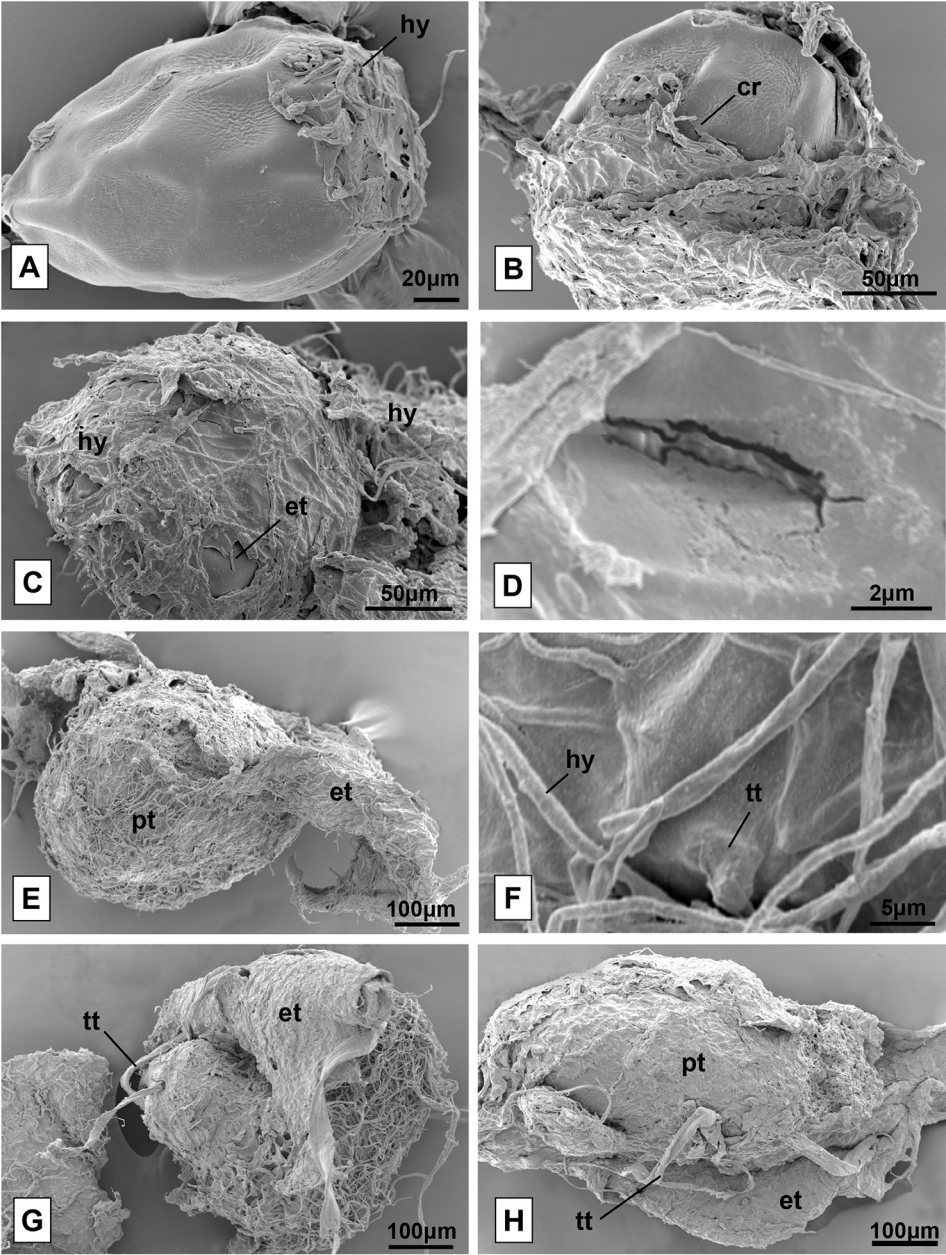
Seeds of *Pogoniopsis schenckii* in co-culture with the isolate F38, under scanning electron microscopy. Seed of *P. schenckii* being infected by fungal hyphae **(A)**. Seeds with cracked external tegument **(B**, **C)** and crack detail **(D)**. Completely cracked external tegument and protocorm exposed and covered by hyphae **(E)** and detail of the beginning of tector trichome formation **(F)**. View of the surface of protocorms and tector trichomes already formed **(G**, **H)**. hy, hyphae; cr, crack; et, external tegument; pt, protocorm; tt, tector trichome.

Due to its ability to cause changes in protocorm development after external tegument rupture, different stages of seed development incubated with the isolate F38 (*Clonostachys* sp.) were analyzed by scanning electron microscopy. The seeds have an oval shape and a wavy external tegument. At their initial germination stage, it is possible to observe the proliferation of fungal hyphae exclusively in the funicular region of the seeds (**Figure 5A**). Then, the seeds increase in size and the external tegument ruptures (**Figures 5B–D**). The protocorm is exposed, fully covered by hyphae and the development of tector trichomes begins (**Figures 5E, F**). In this phase, the protocorm continues to increase in size, taking an elongated form, and scarce tector trichomes can be observed (**Figures 5G, H**). The hyphae growth allowed only partial visualization of the vast majority of protocorms, making it difficult to analyze the samples and collect data.

**Figure 6:**
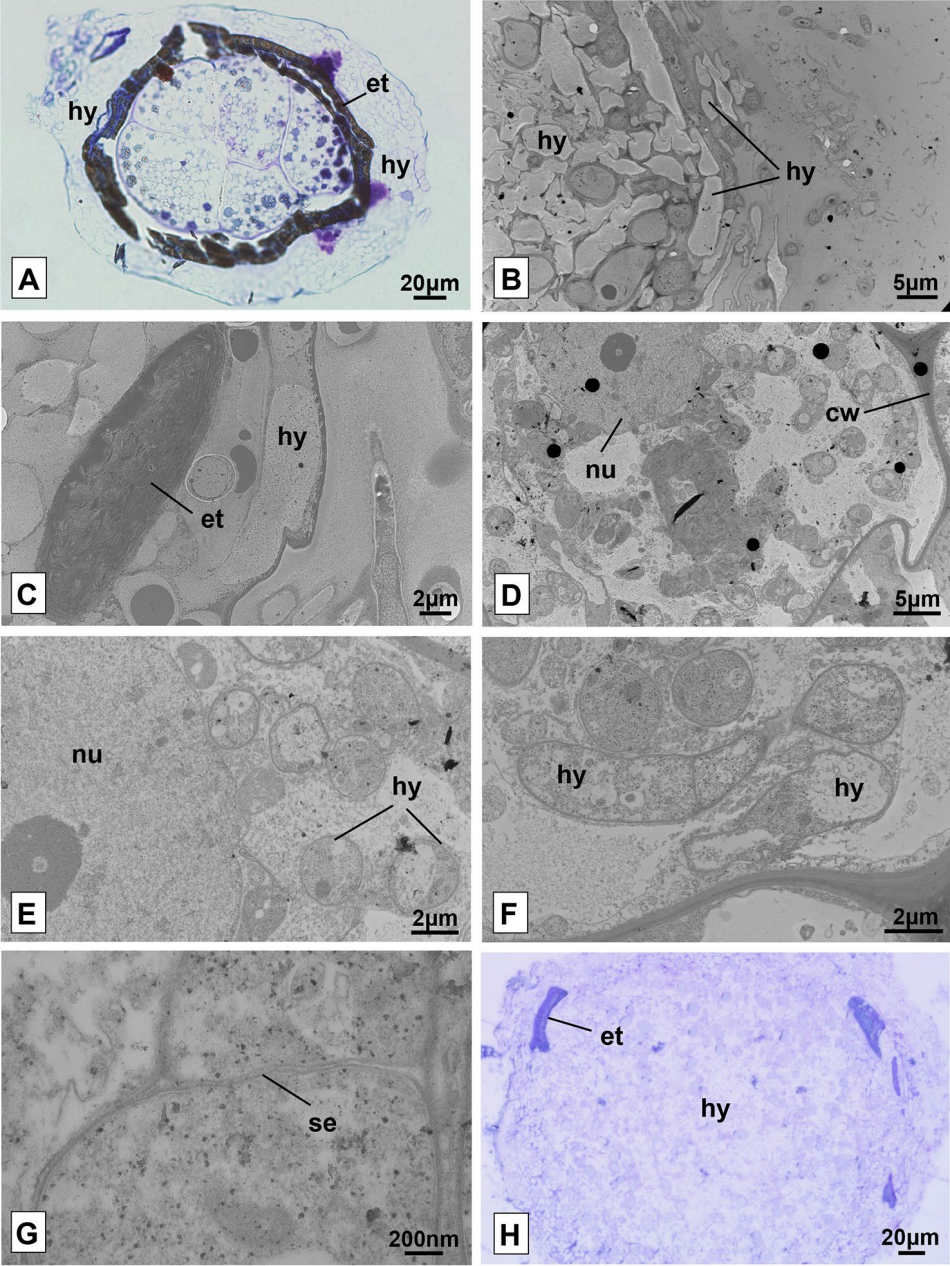
Seeds and protocorms of *Pogoniopsis schenckii* under light and transmission electron microscopy. Seeds showing live cells at stage 1, external tegument scarification, and massive growth of hyphae on its surface after 20 days in co-culture with the isolate F38 **(A)**. Overview of the periphery of the protocorm covered by hyphae **(B)** and detail of fragment of the external integument cracked **(C)**. Integral cell of the protocorm **(D)** and, in detail, cell with evidenced nucleus and hyphae in transverse section **(E)**. Hyphae in longitudinal and transverse section **(F)** and fungal cell septum detail **(G)**. Mass entanglement of hyphae growing from inside the protocorms after phase 5 **(H)**. hy, hyphae; et, external tegument; nu, nucleus; cw, cell wall; se, septum.

The light microscopic analysis of the seeds with the onset of external tegument rupture allowed observing that the plant cells were alive and intact. However, no evidence was found of the formation of pelotons or even the growth of hyphae within the cells at this early stage of germination. The living cells have numerous vacuoles widely distributed in their interior and a large mass of fungal hyphae was also observed on the surface of the tegument (**Figure 6A**).

It was possible to verify the presence of intact plant cells through the analysis of transmission electron microscopy of protocorms resulting from the trials of symbiotic germination at different stages of development. As observed in light microscopy, the surface of the protocorm is covered by a mass of fungal hyphae, and fragments of the ruptured external tegument were found (**Figures 6B, C**). The cells of the observed protocorm had the nucleus and cell wall intact and fungal structures were found occupying much of the interior of the cells, being several times smaller than the first ones (**Figures 6D, E**). The hyphae, surrounded by the membrane of the plant cell (**Figure 6E**), presented several structures with distinct patterns, and some hyphae were filled with dense cytoplasmic content and others with little dense content (**Figure 6F**). It was possible to note the absence of clamp connections, dolipores and parenthesomes in the hyphae found (**Figure 6G**). No formation of pelotonswas observed by the fungal isolate of the genus *Clonostachys*.

After phase 5, the growth of the protocorm was no longer observed, which started being degraded by the fungi. Instead, a cluster of fungal hyphae was found occupying the entire interior of the protocorm and expanding beyond the external tegument of the seeds, forming massive structures (**Figure 6H**). It is believed that at some point in development, protocorm structures were degraded and absorbed by the inoculated fungus, even on plates where protocorms were transferred to new culture media on a monthly basis.

## Discussion

### Endophytic and Mycorrhizal Community

The community of endophytic cultivable fungi of the MH species *P. schenckii* was characterized for the first time. All the fungi isolated from root, floral stem, and fruit belong to the phylum *Ascomycota* and were grouped in the taxon Sordariomycete*s* (Ma et al., 2015). The genera *Xylaria* and *Coniochaeta*, reported for TG population roots, were not found in the plants of TPP and TP populations, which presented only the presence of the genus *Trichoderma*. It is interesting to note that TG population is miles away from the other two, and there is a road separating them geographically. As for floral stem and fruit, the genera found were the same for the two organs, and the genera *Fusarium*, *Clonostachys*, and *Colletotrichum* were isolated. According to Ma et al. (2015), the diversity of non-mycorrhizal endophytic fungi of orchids would be more related to the locality of the orchid collection than to other factors, and it may vary from population to population to the detriment of the geographical differences between them.

The genera *Xylaria*, *Trichoderma*, and *Fusarium* are non-mycorrhizal endophytes often found in association with orchids (Ma et al., 2015). The taxon *Xylariaceae* has been repeatedly found in *Dendrobium* sp. (Yuan et al., 2009; Chen et al., 2011; Chen et al., 2013), and *Xylaria* sp. has also been reported in the green orchid *Anoectochilus setáceus*, from which it was possible to extract an important antibacterial compound (Ratnaweera et al., 2014). Antimicrobial properties have also been observed in *Trichoderma* sp., associated with Cupressaceae (Mahdieh and Soltani, 2014). The abundance of compounds with pharmacological properties found in orchids may have the production, to some extent, related to the great diversity of fungal metabolites present in their tissues, which would represent a benefit to the plant in this association (Ma et al., 2015). The production of metabolites by the endophytic fungi isolated from *P. schenckii* was not investigated in this work, and it is a point to be explored. To our best knowledge, *Coniochaeta* was reported only once in recent work by Lee and Eom (2017), associated with a native orchids from Korea, *Coniochaeta mutabilis*.

Some plant pathogens may appear as asymptomatic endophytes in orchids, such as *Trichoderma*, related to diseases in cotton (Lutfunnessa and Shamsi, 2011), and *Fusarium* (Pecoraro et al, 2013). Although some species of *Colletotrichum* have already been listed as pathogens in orchids such as *Oncidium flexuosum*, *Bulbophyllum cylindraceum* and *Coelogyne cristata* (Tao et al., 2013), they have also been found in healthy tissues of *Lepanthes* and *Dendrobium* (Bayman et al., 1997; Chen et al., 2011) as in *P. schenckii*. In this study, the isolations were only performed in tissues and organs of healthy plants, without any apparent disease.

Differently from what is expected from tropical MH orchids, *P. schenckii* is associated with *Tulasnella* sp., belonging to the groups frequently found for green orchids from temperate regions (Rasmussen, 2002; Rasmussen et al., 2015). Fungi belonging to *Tulasnellaceae* have also been reported previously associated with two orchids of the genus *Neottia* (Těšitelová et al., 2015). For green orchids from the Atlantic Forest, there were identification of fungi belonging to the orders *Sebacinales* and *Cantharellales*, described in Oliveira et al. (2014), in addition to the genera *Ceratorhiza* and *Ephulorhiza* reported in a conservation study conducted by Pereira et al. (2005). The methodology used for the molecular identification of the DNA extracted from the roots of *P. schenckii* is quite specific for *Tulasnellaceae*, bringing into question the fact that other species of fungi may be related to this orchid besides the one reported in the present study. This analysis represented a brief investigation to verify the presence of this taxon, known as being a rhizoctonia associated with the roots of *P. schenckii*, considering the absence of mycorrhizal fungi in the isolation trials by means of culture.

Some genera of mycorrhizal fungi may not be able to grow in culture medium or be isolated through conventional techniques, which may explain their absence among the isolated fungi of *P. schenckii*. In their work, Zelmer et al. (1996) had reported the difficulty of isolating mycorrhizal fungi in *Cypripedium acaule* and *Malaxis monophyllos* and, despite the presence of pelotons in root tissues, no mycorrhizal fungi were isolated in their work. For *P. schenckii*, the absence of typical pelotons was also confirmed for the root cells and subterranean system of the species (unpublished data—Flores-Borges, D.N.A). Although the presence of degraded pelotons cannot be discarded. Moreover, no hyphae were observed with clamp connections, dolipores or parenthesomes, typical characteristics of Basidiomycota (Roy et al., 2009), in fungi of roots (unpublished data—Flores-Borges, DNA), as well as of fruits of *P. schenckii* (unpublished data—Alves, MF). Among the difficult cultivable fungi, there are those not related to rhizoctonia (Zelmer et al., 1996). It was not true for the fungus found in this work, *Tulasnella* sp., which, despite being closely related as the group, as well as Ceratobasidiaceae and Sebacinales, was not possible to be verified by means of the culture dependent identification methodology employed in this study. The PDA medium is very nutrient rich and greatly favors the development of fast-growing fungi, which can lead to the non-development of slower-growing fungi such as Tulasnellaceae (Zettler and Corey, 2018).

### Germination Trials

Regarding the fungi tested, three genera were able to cause seed germination of *P. schenckii*, namely: *Trichoderma*, *Fusarium*, and *Clonostachys*. Among these, only the genus *Clonostachys* stimulated the development of the protocorm beyond the seed testa rupture. *Fusarium* has previously been reported in the literature as being able to cause seed germination of three green orchids, *Cypripedium reginae*, *Cypripedium parviflorum*, and *Platanthera grandiflora* (Vujanovic et al., 2000), and a century earlier, by Bernard (1900, 1903, 1904), qt. in Vujanovic et al. (2000). Other taxa of Ascomycota have also been related to the germination of terrestrial orchid seeds belonging to Neottieae and Orchideae tribes (Rasmussen et al., 2015). According to Vujanovic et al. (2000), *Fusarium* would be able to produce structures very similar to the shape and distribution of monilioid cells, typical of rhizoctonia, and easily confused with them. As far as it is known, there are no reports in the literature about the germination of orchids by fungi of the genus *Trichoderma* or *Clonostachys*.

In preliminary studies by Yagame et al. (2007), it was not possible to induc e the germination of the aclorophyllated orchid *E. roseum* through symbiotic germination in oat meal agar medium despite the use of a mycorrhizal fungus, due to the excessive growth of the fungi in the culture medium, as also reported in the first trials with *P. schenckii*. In subsequent trials, altering oat concentration of the culture medium as well as its renewal by means of periodic transfers of the seeds and protocorms to new plates, it was enough to solve this obstacle. The germination methodology initially cited in this paragraph has been successfully used for several species of green orchids (Stewart and Kane, 2007; Steinfort et al., 2010; Pereira et al., 2015) and, in some cases, for MH ones (Bruns and Read, 2000). The methodology used may not have been adequate to meet the requirements necessary for the complete development of protocorm and establishment of the seedling of this species, or the association with other mycorrhizal fungal taxa may be necessary for this to occur. It is not clear what prevented the later development of the protocorm of *P. schenckii* beyond that reached in this work, and further studies are necessary.

In *in situ* germination experiments by Rasmussen and Whighan (1993), none of the five terrestrial orchid species germinated before 6 months of experiment; two of them, *Liparis liliifolia* and *Tipularia discolor*, were not able to germinate in the 12 months from the beginning of their experiment, and the seeds of *P. schenckii* did not germinate in a period of 18 months. During this period, the seeds remained intermixed by hyphae within the fruits, but without presenting increase in volume or testa rupture (data not shown). As expected, the asymbiotic germination of *P. schenckii* also did not produce results and, to date, no MH orchid species have been able to germinate in the absence of mycorrhizal fungi (Bruns and Read, 2000; McKendrick et al., 2002; Leake et al., 2004; Yagame et al., 2007). Although the *in vitro* symbiotic germination assays show only the initial development of protocorms of *P. schencki*i, and their development is not possible until the establishment of the seedling, this is probably the first report of the germination of a species of heterotrophic and aclorophyllated orchid that is stimulated by the presence of non-mycorrhizal endophytic fungi isolated from roots and, specifically, from fruits.

### Protocorm Development

The mycorrhizal interactions within the Orchidaceae family occur from the association between specific fungal taxa of the phylum Basidiomycota and the orchids (Peterson et al., 2004). This interaction can be verified by the ability of the fungus to cause germination of the plant seeds, besides of its capacity to form pelotons inside the living cells of the plant (Peterson et al., 2004; Rasmussen and Rasmussen, 2009). In this study, despite of causing the germination of the seeds of *P. schenckii*, the formation of pelotons in the cells of the protocorm was not observed for the isolate F38, of the genus *Clonostachys*, being that this fungus does not belong to a taxa known as mycorrhizal. Therefore, the interaction between both cannot be classified as mycorrhizal. However, MH orchids are not known to be able to germinate or even develop independently from a mycorrhizal fungus (Rasmussen and Rasmussen, 2009), and even less in the form of culture to which the seeds of *P. schenckii* were submitted, in culture medium with complex carbon sources (Zettler, 1997). Therefore, it is assumed that some degree of interaction between some of the tested fungal isolates and the orchid protocorms has occurred that seed germination and initial protocorm development was stimulated, although it is not of mycorrhizal nature.

## Conclusion

Identifying fungal endophytes from MH orchids is a key step in understanding species interactions in tropical conditions. All the endophytic fungi present in the roots of *P. schenckii* belonged to the phylum Ascomycota and varied in the detriment of the geographic distance of the populations, as commonly observed for the endophytes of other orchids. The methodology employed for fungal isolation was not able to isolate potentially mycorrhizal fungi from the Tulasnellaceae, found by other means, as well as no other taxon of Basidiomycota.

Differently from what is observed for most of the MH species of tropical regions, *P. schenckii* is associated with fungi commonly found in green orchids. Three different genera of isolated non-mycorrhizal fungi were able to induce the germination of the seeds of the species under the asymbiotic condition and one of them, *Clonostachys* sp., in addition to the external tegument rupture, promoted the initial development of the protocorm. *P. schenkii* protocorms in co-culture with this isolate showed fungal hyphae within living plant cells, but without the formation of typical mycorrhizal pelotons.

*P. schenckii* was not able to germinate in the absence of fungi or in seeding and seed recovery trials *in situ*. This is the first report of stimulation of the germination of the MH orchid species in the presence of non-mycorrhizal endophytic fungi isolated from above and belowground organs of this orchid species.

## Data Availability Statement

All datasets for this study are included in the article/**Supplementary Material**.

## Author Contributions

JM and LS initiated and designed the study. LS carried the anatomical analysis, analysis of scanning microscopy, isolation and analysis of fungi, germination trials and writing the manuscript. MB and LS developed the phylogenetic tree. DF-B carried transmission microscopy and SA and SK assisted molecular analysis of fungi and contributed to the drafts. MB and JM supervised the work and assisted and interpreted of the results.

## Funding

This work was financed by the São Paulo Research Foundation (FAPESP—2015/26479-6), and the National Council for Scientific and Technological Development (CNPq - 447453/2014-9; 310184/2016-9). We thank CNPq and the Coordination for the Improvement of Higher Education Personnel (CAPES) for the master’s degree scholarships to the first author and doctoral scholarship to the second author, respectively. This study was funded in part by CAPES—Finance Code 001.

## Conflict of Interest

The authors declare that the research was conducted in the absence of any commercial or financial relationships that could be construed as a potential conflict of interest.
