# Supplementary material for: The Role of Non-Mycorrhizal Fungi in Germination of the Mycoheterotrophic Orchid Pogoniopsis schenckii Cogn
Source: Front Plant Sci. 2019 Nov 29;10:1589. doi: 10.3389/fpls.2019.01589 (PMC6896934; doi:10.3389/fpls.2019.01589)
Supplement: Supplementary file 1 [file Table_1.docx]

Table 1: Identification of endophytic fungi isolated from *Pogoniopsis schenckii*.

| Fungal Isolated | Genera | Organ of Origin | Population of Origin | Collect Date |
| --- | --- | --- | --- | --- |
| F04 | Xylaria | Root | TG | 08/12/2015 |
| F05 | Xylaria | Root | TG | 08/12/2015 |
| F06 | Xylaria | Root | TG | 08/12/2015 |
| F07 | Xylaria | Root | TG | 08/12/2015 |
| F08 | Xylaria | Root | TG | 08/12/2015 |
| F09 | Xylaria | Root | TG | 08/12/2015 |
| F10 | Coniochaeta | Root | TG | 08/12/2015 |
| F11 | Coniochaeta | Root | TG | 08/12/2015 |
| F13 | Coniochaeta | Root | TG | 08/12/2015 |
| F14 | Coniochaeta | Root | TG | 08/12/2015 |
| F15 | Xylaria | Root | TG | 08/12/2015 |
| F16 | Trichoderma | Raiz | TPP | 31/01/2016 |
| F17 | Trichoderma | Root | TPP | 31/01/2016 |
| F18 | Trichoderma | Root | TPP | 31/01/2016 |
| F19 | Trichoderma | Root | TPP | 31/01/2016 |
| F23 | Trichoderma | Root | TP | 31/01/2016 |
| F24 | Trichoderma | Root | TP | 31/01/2016 |
| F25 | Trichoderma | Root | TP | 31/01/2016 |
| F26 | Trichoderma | Root | TP | 31/01/2016 |
| F27 | Trichoderma | Root | TP | 31/01/2016 |
| F28 | Trichoderma | Root | TP | 31/01/2016 |
| F30 | Trichoderma | Root | TP | 31/01/2016 |
| F31 | Trichoderma | Root | TP | 31/01/2016 |
| F32 | Trichoderma | Root | TP | 31/01/2016 |
| F33 | Trichoderma | Root | TP | 31/01/2016 |
| F34 | Fusarium | Fruit | TPP and TP | 26/03/2016 |
| F35 | Fusarium | Fruit | TPP and TP | 26/03/2016 |
| F37 | Fusarium | Fruit | TPP and TP | 26/03/2016 |
| F38 | Clonostachys | Fruit | TPP and TP | 26/03/2016 |
| F39 | Clonostachys | Fruit | TPP and TP | 26/03/2016 |
| F40 | Fusarium | Fruit | TPP and TP | 26/03/2016 |
| F41 | Colletotrichum | Fruit | TPP and TP | 26/03/2016 |
| F42 | Colletotrichum | Fruit | TPP and TP | 26/03/2016 |
| F43 | Clonostachys | Fruit | TPP and TP | 26/03/2016 |
| F45 | Colletotrichum | Fruit | TPP and TP | 26/03/2016 |
| F46 | Colletotrichum | Fruit | TPP and TP | 26/03/2016 |
| F48 | Clonostachys | Fruit | TPP and TP | 26/03/2016 |
| F49 | Colletotrichum | Fruit | TPP and TP | 26/03/2016 |
| F51 | Fusarium | Fruit | TPP and TP | 26/03/2016 |
| F52 | Colletotrichum | Fruit | TPP and TP | 26/03/2016 |
| F53 | Colletotrichum | Floral stem | TPP and TP | 26/03/2016 |
| F55 | Fusarium | Floral stem | TPP and TP | 26/03/2016 |
| F58 | Fusarium | Floral stem | TPP and TP | 26/03/2016 |

Legend: Trilha do Poço do Pito (TPP), Trilha do Pirapitinga (TP), and Trilha do Garcez (TG).
